# MAGEB16 as an epigenetic timing regulator linking X-chromosome biology to neurodevelopmental vulnerability in Autism Spectrum Disorder

**DOI:** 10.17179/excli2026-9338

**Published:** 2026-03-06

**Authors:** John Antonydas Gaspar, Krishnan M. Dhandapani, David C. Hess

**Affiliations:** 1 Department of Neurology, Medical College of Georgia, Augusta University, Augusta, Georgia, United States; 2 Department of Neurosurgery, Medical College of Georgia, Augusta University, Augusta, Georgia, United States

**Keywords:** MAGEB16, Autism Spectrum Disorder, DNA methylation, neurodevelopmental disorders, epigenetics, X-chromosome, developmental timing, epigenetic switch

## Abstract

MAGEB16 (Melanoma-associated antigen B16) is an X-linked cancer-testis antigen belonging to the MAGE-B family, whose expression is tightly regulated by a promoter DNA-methylation switch that restricts transcription primarily to the male germ line under normal physiological conditions. In addition to its established roles in spermatogenesis and oncogenesis, emerging functional, epigenomic, and genetic evidence points to MAGEB16 as an epigenetically sensitive modifier of early developmental programs implicated in neurodevelopmental disorders such as Autism Spectrum Disorder (ASD). In this study, we performed an integrative analysis combining MAGEB16's chromosomal context, molecular interaction networks, and methylation-dependent regulatory features, alongside experimental depletion datasets from pluripotent stem cells, perinatal cord-blood methylome data from ASD cohorts, peripheral transcriptomics linked to neuropsychiatric risk and recently reported genetic variant associations. Our synthesis identifies underlying evidence indicating that MAGEB16 participates in epigenetically regulated lineage specification processes during early embryonic development. We propose a unified model in which MAGEB16 acts as a dosage- and timing-dependent regulator of early lineage commitment. Disruption of its epigenetic control, particularly during X-chromosome-enriched developmental periods, may influence neurodevelopmental pathways toward ASD-associated phenotypes. These findings position MAGEB16 as a candidate epigenetic-susceptibility factor linking germline-restricted regulatory changes, that could influence early brain development and increase the risk for neurodevelopmental conditions.

See also the graphical abstract(Fig. 1).

## 1. Introduction

Autism Spectrum Disorder (ASD) encompasses a highly heterogenous group of neurodevelopmental differences, not one single disease, that affects behavioral, communicative and learning patterns. One striking feature of ASD is that it affects males more than females. This immediately brings attention to the X chromosome. Investigation into the causative agents revealed a multigenic etiology, with epigenetic regulation playing a critical role. Another important point is timing. Recent work suggests that ASD risk may begin very early, possibly during embryonic or perinatal stages, prior to neuronal maturation. Thus, genes that act developmentally early, even before neural differentiation, may play a central role in shaping ASD risk (Courchesne et al., 2020[3]).

ASD should be considered along a developmental trajectory, not just a diagnosis defined by behavior later in life. Many of the biological events that shape ASD risk likely happen long before symptoms are visible. By the time social or communication differences are observed, the underlying developmental pathways may already be established. Moreover, ASD risk does not appear to come from a single disrupted pathway. Instead, subtle shifts across multiple early programs, including cell fate decisions, neuronal migration, axon guidance, and timing of differentiation, may produce cumulative effects on phenotype (Parikshak et al., 2013[14]; Satterstrom et al., 2020[15]). ASD genetics also shows that many risk genes are involved in regulation rather than structure. These include chromatin modifiers, transcriptional regulators, and epigenetic control proteins (de la Torre-Ubieta et al., 2016[4]). Therefore, ASD risk may be a consequence of when and where gene expression occurs, as opposed to complete loss or gain of gene function (Courchesne et al., 2020[3]).

The male bias in ASD further points toward X-chromosome sensitivity during development that is unlikely to be explained by hormone effects alone. Instead, epigenetic regulation of X-linked genes, during early development may create windows of vulnerability that differ between males and females (Mordaunt et al., 2020[13]). This may include epigenetically controlled, X-linked genes that are not classically associated with neuronal development.

One interesting candidate gene, MAGEB16, is a cancer-testis antigen (CTA). CTAs are a class of genes normally expressed in germline tissues and early developmental stages but epigenetically silenced in most somatic tissues; they can be aberrantly reactivated in cancer (Doyle et al., 2010[5]; Lee and Potts, 2017[10]). The molecular role of MAGEB16 was first delineated in the scientific literature as a pluripotency-associated factor that supports the maintenance of the undifferentiated state in pluripotent stem cells (Gaspar et al., 2012[6]). Moreover, MAGEB16 has been studied in spermatogenesis and tumorigenesis, but recent functional and epigenomic data point to a broader relevance in early developmental programming (Gaspar et al., 2017[7]; Mordaunt et al., 2020[13]). MAGEB16 may therefore represent an epigenetically gated developmental modifier whose mistiming sensitizes early neurodevelopmental programs. This mini-review will summarize what is known about MAGEB16, including potentially unexplored links to ASD.

## 2. Gene Biology and Chromosomal Context of MAGEB16

### 2.1 Human MAGEB16

Human MAGEB16 is found on the short arm of the X chromosome, in a region called Xp21.1, which contains many genes in the MAGE-B family (from MAGEB1 to MAGEB18) (Liu et al., 2014[12]; Lee and Potts, 2017[10]). These genes are grouped together in one block that spans several million base pairs. The fact that this cluster is conserved through evolution suggests an important biological function. This same region sits very close to other clinically important genes, including DMD, GK, and NR0B1 (also known as DAX1), which are involved in muscle, metabolic, and endocrine disorders. Nearby, there are also other cancer-testis antigen gene clusters, such as MAGE-C and XAGE. Many of the genes in this wider neighborhood share similar behavior: they are usually active only in the testis and are often not fully silenced by X-chromosome inactivation. This means they are unusually sensitive to epigenetic regulation (Gupta et al., 2019[9]).

**Figure 2:**
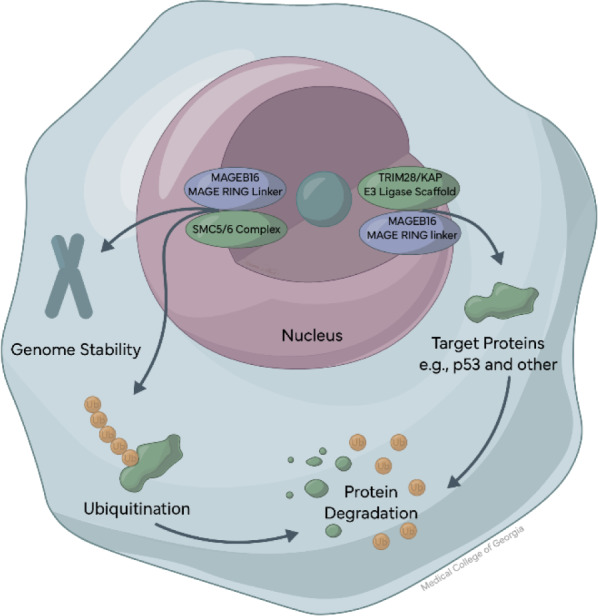
MAGEB16 likely functions as an adaptor protein recruiting TRIM28/KAP1 ubiquitin ligase complexes and the SMC5/6 complex, thereby influencing protein stability, chromatin regulation, genome maintenance and protein degradation.

Studies of MAGE-B genes indicate involvement in basic cell survival functions, especially in germ cells, including roles in cellular division, programmed cell death, and stress responses. Although the precise mechanisms remain undefined, MAGE-B proteins interact with protein complexes that control protein degradation, particularly E3 ubiquitin ligases such as TRIM28/KAP1, as well as the SMC5/6 complex, which is involved in maintaining genome stability (Figure 2(Fig. 2)) (Doyle et al., 2010[5]; Lee and Potts, 2017[10]).

In cancer, this entire region often becomes abnormally hypomethylated, meaning the normal epigenetic “locks” that keep these genes silent are removed. When this happens, many MAGE-B genes are simultaneously activated. Because of this coordinated behavior, the Xp21.1 region can be thought of as an epigenetic control hub. When its regulation is disturbed, germline-specific gene programs that should remain silent can be reactivated inappropriately, contributing to oncogenesis (Lee and Potts, 2017[10]).

### 2.2 Mouse Mageb16

The mouse version of Mageb16 is located in the XF3 region of the X chromosome (around chrX: 97.46 Mb), which also contains an expanded cluster of related Mage-b genes (Mageb1-Mageb18) that closely mirrors the organization seen in humans (Liu et al., 2014[12]). Like the human gene, mouse Mageb16 shows strict testis-specific expression, and this pattern is controlled by DNA methylation at its promoter. When the promoter is methylated, the gene is silent; when demethylated, the gene becomes active, showing that the basic regulatory mechanism is conserved across species. Studies of mouse Mage-b gene reveal key roles in meiosis, genome stability, and germ-cell survival that are reliant on interactions with TRIM28 and the SMC5/6 complex, particularly involving NSMCE3, which is itself a MAGE family member (Atkins et al., 2020[1]). This suggests that Mageb16 is part of a broader, conserved molecular network rather than acting alone.

Importantly, functional insight into Mageb16 comes from pluripotent stem-cell studies, where depletion of Mageb16 does not block cell survival but instead accelerates exit from pluripotency and biases differentiation toward mesodermal lineages (Gaspar et al., 2017[7]). This finding indicates that Mageb16 influences developmental timing and lineage balance, rather than acting as a germ-cell-restricted factor only. Taken together, the conserved epigenetic control, nuclear localization, shared interaction partners, and stem-cell phenotypes strongly support functional orthology between mouse and human Mageb16, while also pointing to a broader role in early developmental regulation. Despite a lack of Mageb16 knockout data, studies of related Mage-b genes in mice show roles in genome stability and germ-cell survival. This supports the idea that the functions of Mageb16 are fundamental and conserved.

## 3. Molecular Interactions and Epigenetic Control

### 3.1 Cellular localization and molecular function

MAGEB16 is mainly localized within the nucleus, overlapping sites of gene regulation. Rather than acting as a direct regulator of gene transcription, MAGEB16 likely works indirectly, by influencing the stability or availability of key regulatory proteins, based on direct evidence and strong inference from closely related MAGE-B family members. The presence of MAGEB16 in discrete nuclear foci, together with proteins like TRIM28/KAP1 and components of the SMC5/6 complex, suggests that MAGEB16 may be involved in maintaining chromatin stability during periods of cellular stress or rapid division (Doyle et al., 2010[5]; Brattås et al., 2017[2]). Through its role as an adaptor for E3 ubiquitin ligase complexes, MAGEB16 may help determine which proteins are removed and when, especially proteins involved in DNA damage responses and cell-cycle control (Doyle et al., 2010[5]; Lee and Potts, 2017[10]). In this way, MAGEB16 may support cell survival while also shaping the timing of developmental transitions.

### 3.2 DNA methylation as a binary regulatory switch

The strict DNA methylation control of MAGEB16 makes it unusual compared to many developmentally expressed genes, which often show gradual or tissue-specific regulation. Instead, MAGEB16 behaves more like a binary system, where relatively small changes in promoter methylation can completely silence or activate the gene. In somatic tissues, dense CpG island methylation enforces transcriptional silencing, whereas in spermatogonia and primary spermatocytes, targeted demethylation permits expression (Liu et al., 2014[12]).

This type of regulation is particularly relevant during early development, when global epigenetic remodeling occurs. During these periods, even brief or partial loss of methylation at the MAGEB16 promoter could allow transient or mistimed expression, which may be sufficient to influence downstream developmental programs. This mechanism is conserved across species and recapitulated in disease states: global hypomethylation in cancer reactivates MAGEB16, while hypermethylation in TET-mutant lymphomas leads to its silencing (Liu et al., 2017[11]). The recurrence of this same methylation-based control in both normal germline biology and disease states reinforces the idea that MAGEB16 functions as a sensitive readout of epigenetic state, rather than a constitutively active developmental gene.

## 4. MAGEB16’s Functional Evidence from Pluripotent Stem Cells

Mageb16 depletion in pluripotent stem cells, via shRNA-mediated depletion, led to an accelerated exit from pluripotency and preferential differentiation toward mesodermal lineages (Gaspar et al., 2017[7]). This finding is critical because pluripotent stem cells represent a very early developmental state, before neural commitment. Thus, a developmental shift in lineage commitment could greatly impact subsequent brain development.

**Figure 3:**
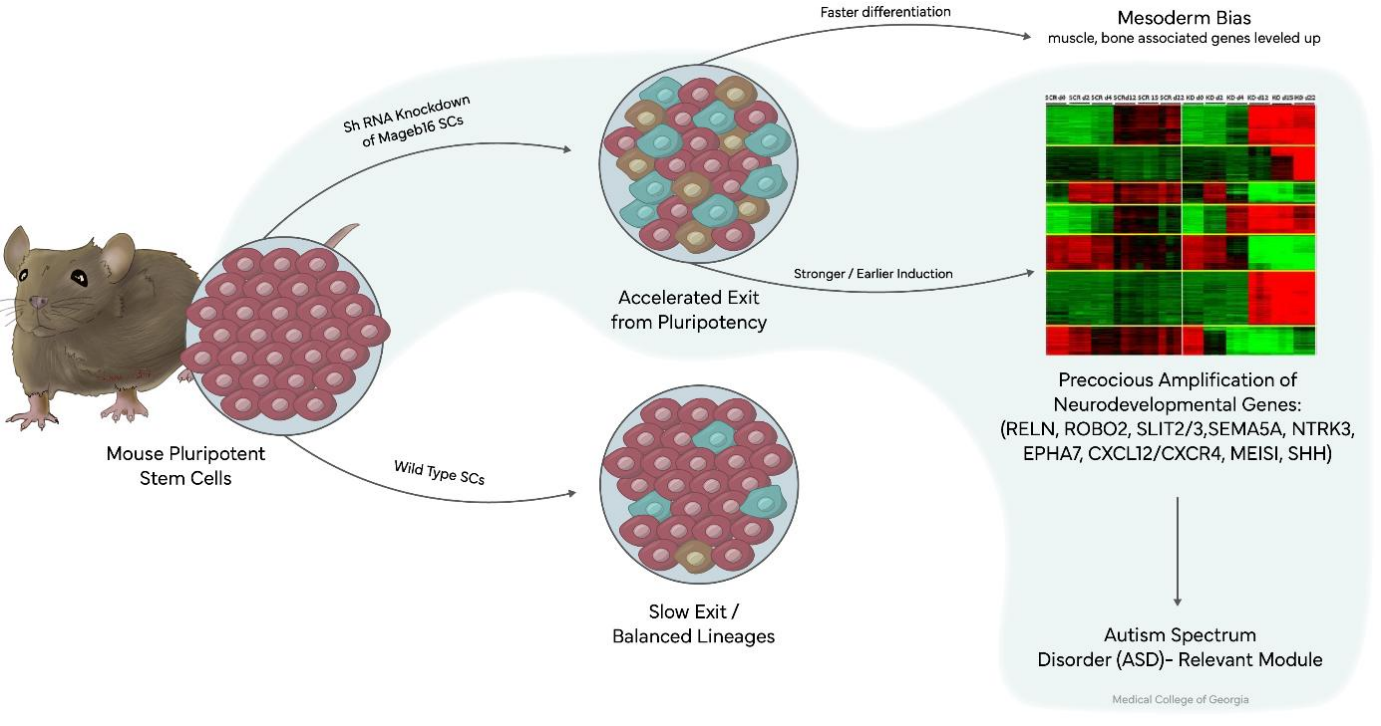
Time-course transcriptomic analysis following shRNA-mediated Mageb16 depletion in pluripotent stem cells. Differential gene expression across defined post-depletion time points reveals accelerated exit from pluripotency and premature activation of neurodevelopmental and axon guidance gene networks associated with Autism Spectrum Disorder.

Time-course transcriptomic analysis studies reveal that Mageb16 depletion does not cause death or random loss of cell identity. This suggests Mageb16 is a regulatory buffer, rather than a housekeeping gene. More specifically, Mageb16 appears to act as a brake, as loss of Mageb16 accelerates and redirects developmental processes. Unsupervised clustering of differentially expressed genes between wild-type and Mageb16 depleted cells show a specific gene signature, with enriched genes involved in axonal guidance, neuronal migration, early patterning, and developmental signaling gradients (Figure 3(Fig. 3)). Many of these differentially regulated genes following Mageb16 depletion have been repeatedly implicated in ASD studies, including RELN, ROBO2, SLIT2/3, SEMA5A, NTRK3, EPHA7, CXCL12/CXCR4, MEIS1, BCL11B, SHH, ID4 and NR2F2. While not functionally implicated in synaptic function, these genes are broadly related to guidance, positioning, and early network formation. This aligns extremely well with the idea that ASD risk arises from early mis-patterning, not late synaptic failure alone. Importantly, these genes were not simply upregulated. Rather, these were activated earlier than normal and with greater amplitude. This finding suggests Mageb16 normally helps keep these programs *in check* until the correct developmental moment.

So, in simple terms explaining when Mageb16 is present at right dosage level, developmental programs unfold on schedule; however, when Mageb16 is reduced, the developmental programs start too early. This is exactly what one would expect from an epigenetically gated timing regulator. Mageb16 is not itself a transcription factor for these genes, but the above finding suggests its effect is indirect, likely involving chromatin organization, protein stability (via ubiquitination pathways), and interaction with TRIM28 and SMC5/6 complexes. Thus, Mageb16 may shape the chromatin environment that controls when these large developmental modules become accessible. This stem-cell evidence is the cleanest demonstration that Mageb16 affects developmental timing, not just expression level.

## 5. MAGEB16’s Associations with Neurodevelopmental and Psychiatric Phenotypes

### 5.1 Perinatal epigenomics in ASD

Cord-blood methylation studies are a powerful tool that captures a snapshot close to birth, before postnatal experience reshapes biology. As Mordaunt et al. (2020[13]) reported in their data set, one of the strongest patterns is X-chromosome enrichment among differentially methylated regions (DMRs). This immediately fits with the male bias in ASD. Whole-genome bisulfite sequencing of cord blood from newborns later diagnosed with ASD has revealed X-chromosome-enriched differentially methylated regions (DMRs) targeting neurodevelopmental and immune pathways. MAGEB16 appears as one of the replicated DMRs, notable for its novelty rather than overlap with prior ASD gene lists. While locus-specific effect sizes are modest, convergence at the level of X-chromosome epigenetic dysregulation supports MAGEB16 as a hypothesis-generating candidate within a broader developmental signature. The effect size is not large, but that is expected. ASD is polygenic and poly-epigenetic. In complex disorders, replication and biological plausibility matters more than raw effect size. What makes MAGEB16 interesting disease modifying candidate is, expression is normally silent due to tight methylation control in somatic tissues; thus, small methylation changes may have binary consequences.

Even partial relaxation of methylation at this locus could allow leaky or transient expression at a developmentally inappropriate time, regardless of absolute expression levels. Even brief or low-level expression during a sensitive window could shift developmental trajectories. Also, the fact that MAGEB16 was not previously known as an ASD gene strengthens the argument that ASD risk involves developmental regulators, not just canonical neural genes. Therefore, the cord-blood data support the idea that epigenetic disruption occurs early, the X chromosome is particularly involved and MAGEB16 sits within this vulnerable epigenetic landscape. This fits well with the stem-cell timing model reported by Gaspar et al. (2017[7]).

### 5.2 Peripheral transcriptomics in psychosis risk

At first glance, blood-based gene expression might seem irrelevant to brain development. But repeatedly, immune and developmental genes show up in psychiatric risk studies. In the ultra-high-risk psychosis RNA-seq study, MAGEB16 was the only gene consistently differentially expressed, detected across multiple normalization pipelines applied in filtering (Goh et al., 2017[8]). That is very striking. This does not mean MAGEB16 causes psychosis as a canonical psychosis gene. Instead, this likely means MAGEB16 is a marker of a broader systemic program, possibly related to stress response, immune-developmental crosstalk and epigenetic instability. Cancer-testis antigens (CTAs) are known to behave this way. They often appear when epigenetic repression is loosened. This would help us to understand thorough this RNA-seq study, neurodevelopmental disorders are not brain-only and peripheral signals can reflect early developmental history. When it comes to MAGEB16 expression in blood, these data may help to understand this as a residual echo of early epigenetic states and marker of chromatin regulation stress. This would strengthen the indication that Mageb16-related pathways operate system-wide, not just in neurons.

### 5.3 Genetic association signals

Machine-learning-based analyses of ASD cohorts initially ranked MAGEB16 among higher-priority X-linked contributors based on aggregate patterns of common variation; although these models were later retracted due to methodological limitations, their findings remain relevant when viewed alongside simpler statistical evidence (Wang and Avillach, 2021[16]). Notably, chi-square-based variant enrichment at the MAGEB16 locus persists, suggesting that its emergence was not solely an artifact of complex modeling. This is particularly important given the challenges of analyzing X-linked variation in ASD, where sex-specific effects and inconsistent handling of the X chromosome can dilute true biological signals. Importantly, the modest and context-dependent nature of these genetic findings aligns well with perinatal epigenomic data showing early X-chromosome methylation differences in ASD (Section 5.1) and with peripheral transcriptomic evidence implicating MAGEB16 as a marker of broader developmental or stress-response programs (Section 5.2). Taken together, these converging lines of evidence support a model in which MAGEB16 does not act as a primary ASD risk gene, but rather as a context-dependent, epigenetically gated developmental modifier, whose subtle genetic effects may become biologically meaningful only during sensitive early developmental windows.

## 6. Integrative Model: MAGEB16 as a Developmental Modifier

Collectively, the available molecular, epigenomic, and developmental evidence supports a model in which MAGEB16 functions as an epigenetically gated modifier of early lineage timing. In this framework, perinatal or early embryonic epigenetic perturbations, particularly those affecting X-chromosome methylation, alter MAGEB16 dosage or timing, leading to premature or exaggerated activation of neurodevelopmental gene networks. Such dysregulation could lead to premature or exaggerated activation of neurodevelopmental gene networks, mirroring the transcriptomic accelerations observed following Mageb16 depletion in pluripotent stem cells (Gaspar et al., 2017[7]) and align with ASD-associated perinatal epigenomic signatures (Mordaunt et al., 2020[13]).

Importantly, current functional evidence derives predominantly from a single shRNA-mediated depletion study in pluripotent stem cells. While these findings strongly suggest a developmental timing phenotype, RNA interference approaches are susceptible to incomplete silencing and potential off-target effects. Independent validation using CRISPR/Cas9-mediated knock-out or locus-specific editing strategies, ideally combined with rescue experiments restoring MAGEB16 expression, will be essential to confirm specificity. Moreover, extending this investigation to human iPSC-derived neural progenitors and early neuroectodermal models will be critical to determine whether MAGEB16 dysregulation directly alters neural lineage commitment, proliferation kinetics, or differentiation timing in a neurodevelopmentally relevant context.

MAGEB16's genomic location further strengthens in relevance to ASD biology. The gene resides within Xp21.1, a region containing several MAGE-B family members that exhibit partial or variable escape from X-chromosome inactivation in females. This escape may generate mosaic and dosage-variable expression patterns in females, potentially safeguarding against full dosage alterations and contributing to reduced ASD predominance in females compared to hemizygous males. This genomic feature supports a threshold model in which epigenetic dysregulation (e.g., partial promoter hypomethylation) must exceed a critical dosage boundary to permit sufficient leaky or mistimed expression capable of shifting early developmental programs towards ASD-associated phenotypes. This “dosage and context dependence” could explain why MAGEB16 emerges as a subtle, rather than dominant, modifier in genetic and epigenetic association studies. Such a model may also help reconcile male predominance in ASD with more variable prevalence in females.

MAGEB16 belongs to a family of proteins implicated in genome stability, ubiquitin-mediated protein turnover, and chromatin regulation through interaction with TRIM28/KAP1 and SMC5/6 complexes. Given these functions, MAGEB16 dysregulation may exert pleiotropic developmental effects extending beyond neurodevelopment. Early organogenesis processes, including placental development, cardiac morphogenesis and extraembryonic tissue regulation, are particularly sensitive to epigenetic instability. Further studies should therefore examine MAGEB16 expression and methylation dynamics in ethically sourced placental tissue, stem cell-derived developmental models and relevant animal systems to assess whether its dysregulation contributes to broader developmental vulnerability rather than exclusively brain-specific outcomes.

Peripheral transcriptomic findings linking MAGEB16 expression to psychosis risk require cautious interpretation. Cancer-testis antigens (CTAs) are known to respond to systemic stress responses, immune activation, inflammatory signaling, or broader epigenetic instability rather than primary neurodevelopmental programming. Longitudinal studies together with single-cell and cell-type specific analyses, will be necessary to distinguish developmental dysregulation from reactive or secondary expression changes.

## 7. Conclusions and Future Evaluations

This mini review proposes that MAGEB16 represents a biologically plausible epigenetic susceptibility factor linking X-chromosome biology with early developmental timing mechanisms relevant to Autism Spectrum Disorder. While existing functional data remain preliminary and largely derived from pluripotent stem-cell models, they consistently support a role in modulating developmental tempo rather than serving as a neuronal gene.

**Figure 4:**
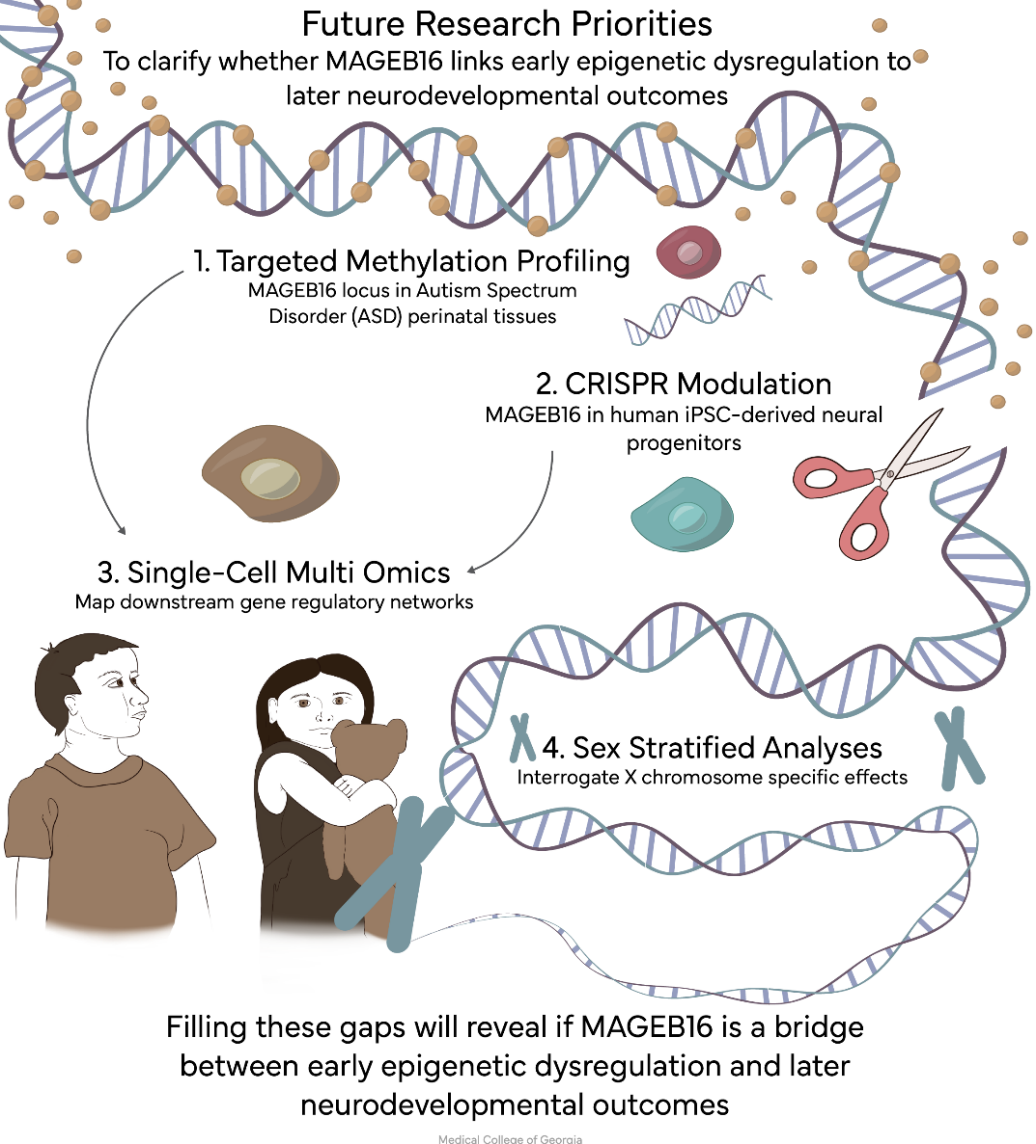
Proposed studies include locus-specific methylation profiling, CRISPR-based functional modeling in neural progenitors, single-cell multi-omics, and sex-stratified analyses of X-linked developmental effects.

Future research should focus on prioritizing CRISPR-based knockout, knock-in, and rescue experiments to validate locus specific function and exclude off-target RNA interreference effects. Functional modeling in human iPSC-derived neural progenitors would define effects on neural lineage specification and developmental timing. Locus-specific methylation profiling in perinatal tissues from ASD cohorts, with sex-stratified analyses addressing X-inactivation escape dynamics, would answer the question of male predominance. Single cell multiomics approaches may assist in mapping downstream chromatin and transcriptional networks influenced by MAGEB16 (Figure 4(Fig. 4)). Ethically viable developmental tissue profiling, including extraembryonic and organogenesis contexts can be employed to evaluate potential pleiotropic roles. Addressing these gaps will clarify whether MAGEB16 constitutes a mechanistic bridge between early epigenetic dysregulation and later neurodevelopmental outcomes.

## Declaration

### Conflict of interest

The authors declare that they have no conflict of interest.

### Artificial Intelligence (AI) - assisted technology

AI was not used to generate scientific ideas, interpret data, or develop the conceptual content of the manuscript except for improving language flow and grammar.

### Special acknowledgment

Ms. Shannon Derthick, M.Sc., helped in designing the figures.

## Figures and Tables

**Figure 1:**
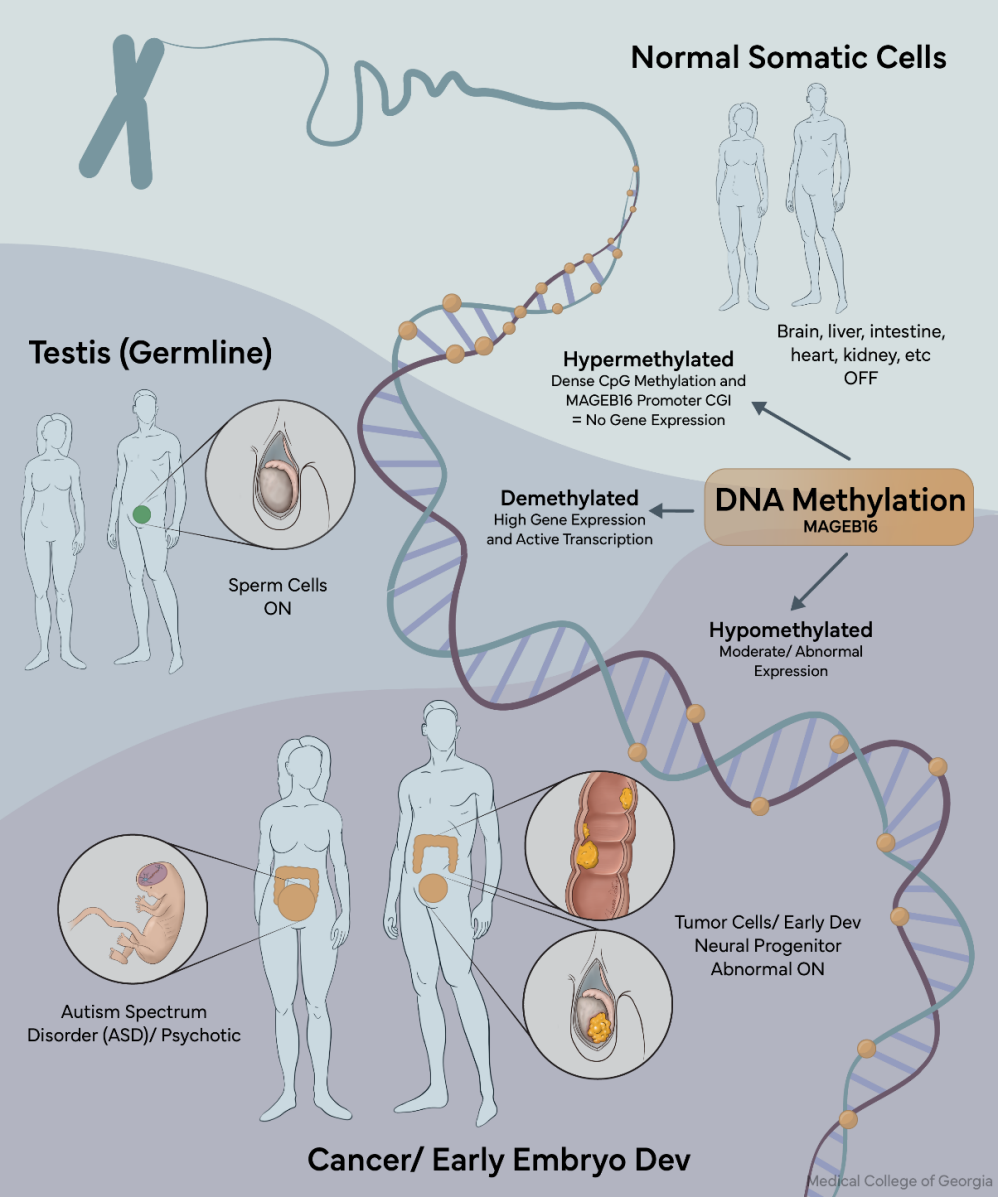
Graphical abstract: Dense promoter methylation silences MAGEB16 in somatic tissues, while targeted demethylation enables physiological expression in germline cells. Pathological hypomethylation results in ectopic activation in cancers such as testicular and colorectal tumors.
